# Supplementary material for: Uniaxial stress flips the natural quantization axis of a quantum dot for integrated quantum photonics
Source: Nat Commun. 2018 Aug 3;9:3058. doi: 10.1038/s41467-018-05499-5 (PMC6076237; doi:10.1038/s41467-018-05499-5)
Supplement: Supplementary file 1 — Supplementary Information [file 41467_2018_5499_MOESM1_ESM.pdf]

# Uniaxial stress flips the natural quantization axis of a quantum dot for integrated quantum photonics

[illegible]

**Yuan *et al*.**

**Supplementary Information**

**Supplementary Note 1: Sample Growth and Structure**

The sample was grown by solid-source molecular epitaxy (MBE) on semi-insulating GaAs (001) substrate. After oxide desorption, a 200 nm thick GaAs buffer was grown at a substrate temperature $T_{sub}$ = 580 °C. Then the substrate temperature was increased to 600 °C and a 100 nm thick $Al_{0.75}Ga_{0.25}As$ sacrificial layer was deposited, followed by 5 nm GaAs, which was used to smoothen the surface and avoid oxidation of the membranes after releasing them from the substrate. Subsequently, 90 nm $Al_{0.2}Ga_{0.8}As$ and 30 nm $Al_{0.4}Ga_{0.6}As$ layers were deposited in sequence. Nanoholes were then etched on the surface of this layer by droplet etching [1,2]: After stopping the arsenic supply, 0.5 monolayer Al were deposited so that the Al droplet can etch holes into the surface of $Al_{0.4}Ga_{0.6}As$ layer during a subsequent annealing step under As flux. To infill the nanoholes, we deposited 1.6 nm GaAs followed by 2 minutes annealing. The GaAs QDs formed in the nanoholes due to the diffusion of GaAs driven by capillarity effects [3]. Since the surface is practically flat after this step [4], the shape of the QDs can be assumed to coincide with that of the nanoholes. An inverted AFM image of such a nanhole is shown in Fig. 1(a) of the main text. The QDs were capped with 30 nm $Al_{0.4}Ga_{0.6}As$ layer, followed by 90 nm $Al_{0.2}Ga_{0.8}As$ layer. Finally, a 5 nm GaAs completes the growth. The whole structure of the as-grown sample is shown in Supplementary Figure 1.

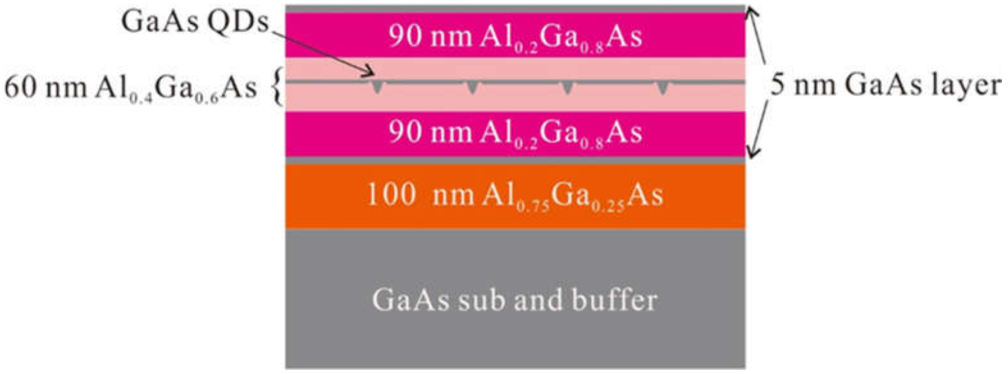


**Supplementary Figure 1**. Structure of the GaAs QDs sample used in this work.

**Supplementary Note 2: Device Processing**

500-µm thick, (001)-oriented, PMN-PT substrates (TRS Techonogies Inc.) were lapped and polished to a thickness of ~300 µm and cut by a commercial 3D-Micromac micromachining system equipped with a femtosecond laser[5,6]. The laser spot was focused down to 5 µm.

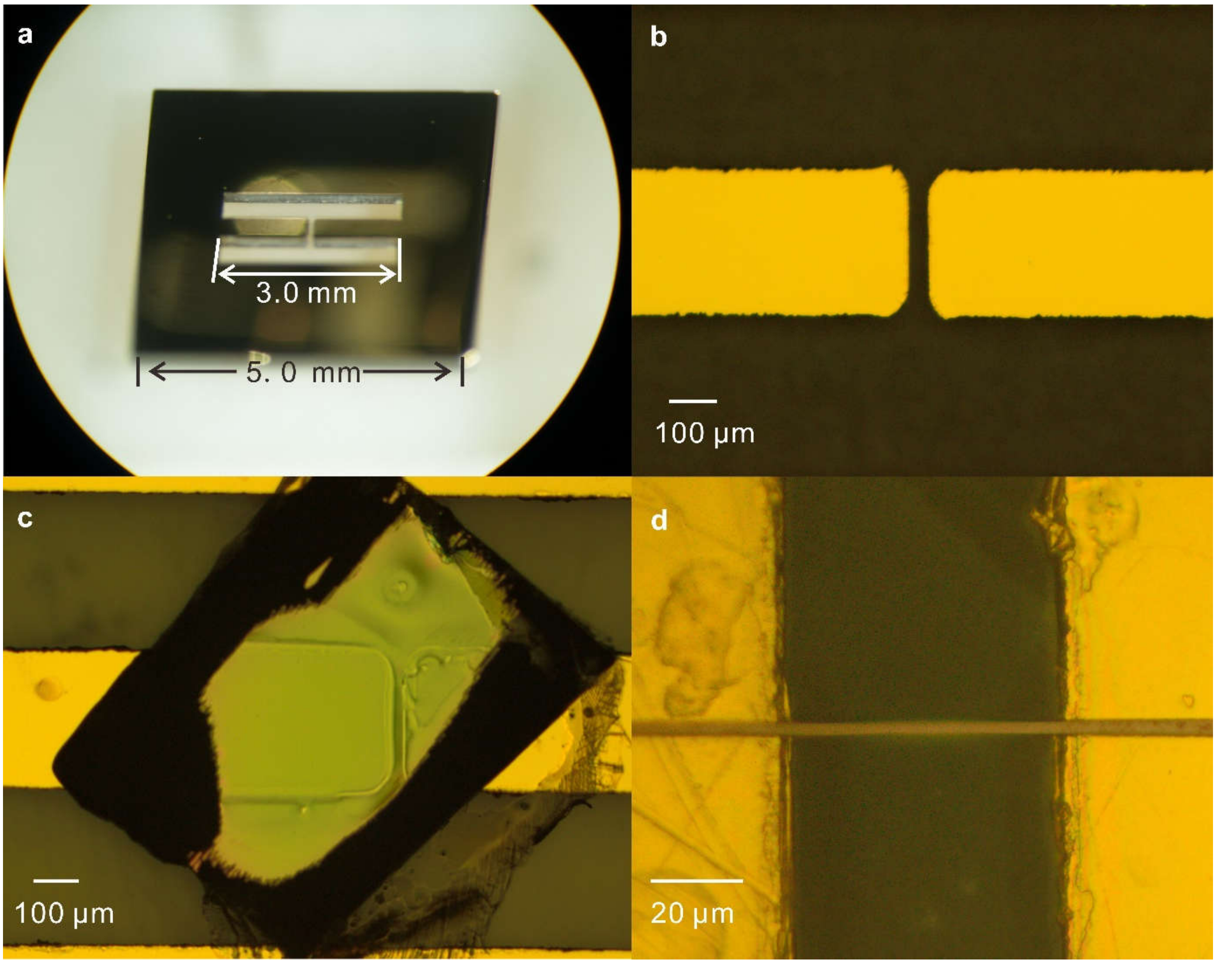


**Supplementary Figure 2**(a), Microscope image of the micro-machined "two fingers" PMN-PT actuator. (b), Enlarged microscope picture of the gap between the two fingers. (c), Micrograph of one device studied in this work, a large QDs-containing membrane bonded on the PMN-PT actuator (d), Microscope picture of another device featuring a narrow stripe membrane (3.5 μm in width ) bonded on the PMN-PT actuator.

A representative picture of a micro-machined "two-fingers" $[Pb(Mg_{1/3}Nb_{2/3})O_3]_{0.72}[PbTiO_3]_{0.28}$ (PMN-PT) actuator employed in our study is shown in Supplementary Figure 2(a). Because the total length of the two fingers (3.0 mm in total) is much larger than the length of the gap (20 ~ 60 μm) the relative size change of the gap is much larger than the relative deformation of the fingers when an electric field $F_p$ is applied across the fingers. This is the key why this novel micro-machined PMN-PT can provide large strain amplification. Supplementary Figure 2(b) shows a microscope image of the gap between the two fingers.

Information on the membrane processing and integration on the actuator can be found in Supplementary Note 5 and in Refs.[5,6]. Supplementary Figure 2(c) and (d) show the two different sizes of membranes which we studied in this work. Supplementary Figure 2(c) presents an optical microscope picture of the device with a large membrane used for the data shown in in Figure 3 in the main text and Supplementary Figure 2(d) shows an image of a device with narrow stripe membranes, used in Figure 2 and Figure 4 in the main text.

**Supplementary Note 3: Stress configuration and finite element simulations**

Since in the experiment we were not able to provide an independent measurement of the strain configuration produced by the piezoelectric actuator, we have performed finite element method (FEM) simulations. We provide below the relation between stress and strain in the case of ideal uniaxial stress along the [100] crystal direction of GaAs and the results of the numerical calculations.

Using the Voigt notation for the stress and strain tensors, the Hook's law for a cubic semiconductor reads:

$$\begin{pmatrix} \sigma_{xx} \\ \sigma_{yy} \\ \sigma_{zz} \\ \sigma_{yz} \\ \sigma_{xz} \\ \sigma_{xy} \end{pmatrix} = \begin{pmatrix} C_{11} & C_{12} & C_{12} & 0 & 0 & 0 \\ C_{12} & C_{11} & C_{12} & 0 & 0 & 0 \\ C_{12} & C_{12} & C_{11} & 0 & 0 & 0 \\ 0 & 0 & 0 & C_{44} & 0 & 0 \\ 0 & 0 & 0 & 0 & C_{44} & 0 \\ 0 & 0 & 0 & 0 & 0 & C_{44} \end{pmatrix} \begin{pmatrix} \varepsilon_{xx} \\ \varepsilon_{yy} \\ \varepsilon_{zz} \\ 2\varepsilon_{yz} \\ 2\varepsilon_{xz} \\ 2\varepsilon_{xy} \end{pmatrix} \tag{1}$$

Here the $\sigma_{ij}$ are the independent components of the stress tensor, $C_{ij}$ the components of the elastic stiffness tensor and $\varepsilon_{ij}$ the strain tensor. Since in our case the uniaxial stress is applied along the [100] direction, there is no shear stress, so that the above equation can be further simplified as:

$$\begin{pmatrix} \sigma_{xx} \\ 0 \\ 0 \end{pmatrix} = \begin{pmatrix} C_{11} & C_{12} & C_{12} \\ C_{12} & C_{11} & C_{12} \\ C_{12} & C_{12} & C_{11} \end{pmatrix} \begin{pmatrix} \varepsilon_{xx} \\ \varepsilon_{yy} \\ \varepsilon_{zz} \end{pmatrix} \tag{2}$$

$$\varepsilon_{yy} = \varepsilon_{zz} = -\frac{C_{12}}{C_{11}+C_{12}}\varepsilon_{xx} = -\nu_{[100]}\varepsilon_{xx} \tag{3}$$

Here $\nu_{[100]}$ is the Poisson ratio for uniaxial stress along the [100] direction (ν = 0.31 for GaAs at a temperature of about 10 K[7]).

We used the commercial software package COMSOL Multiphysics to perform Finite Element Method (FEM) simulations for our actuators. All calculations assume linear elastic deformations. All the parameters are set based on one of the devices. We should mention that in the simulation we used the elastic and piezoelectric parameters of PMN-PT at room temperature. Since it is known that the piezoelectric constants of PMN-PT will decrease at cryogenic temperature[8], we adjusted the electric field applied to the PMN-PT to obtain strain magnitudes similar to our experiment.

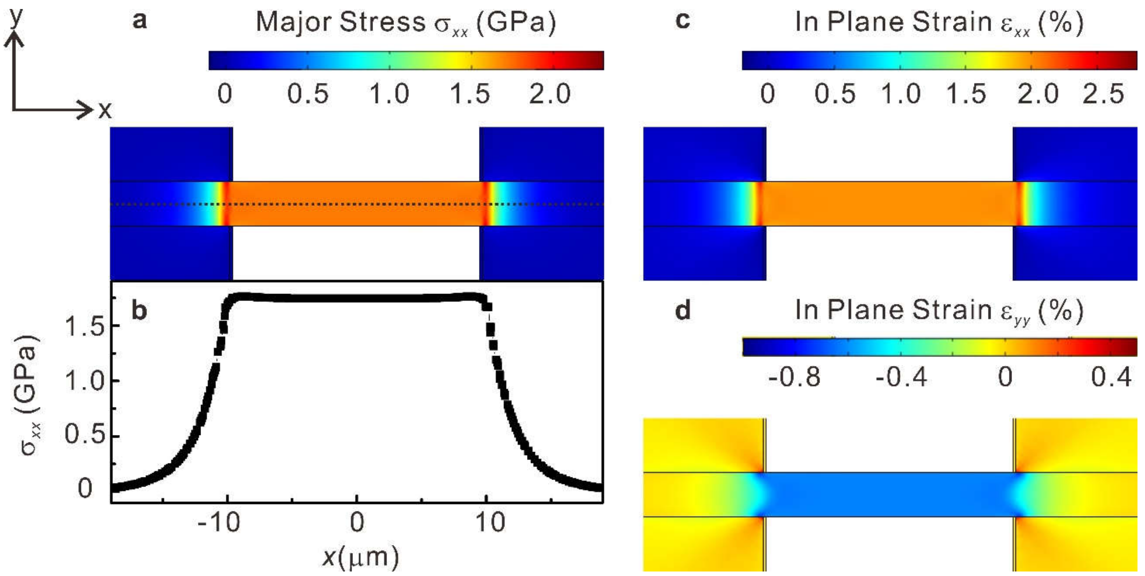


**Supplementary Figure 3**. FEM simulations of the stress and strain distribution in our devices. (a) Major stress $\sigma_{xx}$ map in the middle of the membrane in *z* direction (the same location of the QDs). The dashed line shows the position of the line scan in (b). (c, d) In plane strain ($\varepsilon_{xx}$, $\varepsilon_{yy}$) maps, respectively. We verified that $\varepsilon_{zz}=\varepsilon_{yy}$, consistent with a uniaxial stress configuration.

Supplementary Figure 3(a) shows the major stress $\sigma_{xx}$ map in our device. It shows quite homogeneous tensile stress in the suspended area of the membrane. In order to have a clearer picture of the stress distribution along the *x* direction of the membrane, we make a line scan [the line is locate in the middle of membrane, marked as dashed line in Supplementary Figure 3(a)] along *x*, see Supplementary Figure 3(b). The suspend area (-10 μm < *x* < 10 μm) shows homogeneous tensile stress, while the tensile stress falls rapidly when we move to the area

bonded on the PMN-PT actuator and changes into compressive stress when moving away from the gap (not shown here). As mentioned above, for an ideal uniaxial stress, $\varepsilon_{yy} = \varepsilon_{zz} = -\nu_{[100]}\varepsilon_{xx}$. Supplementary Figure 3(c) and (d) show the in plane strain maps of the strain tensor components $\varepsilon_{xx}$ and $\varepsilon_{yy}$ from the simulation. It is clear that in the suspend area there is tensile strain along $x$ direction ($\varepsilon_{xx}$ = 2.0% > 0) and compressive strain along $y$ direction ($\varepsilon_{yy}$ = -0.62% < 0), the Poisson ratio $\nu_{[100]}$ is about 0.31, consist with the expected value.

The impact of stress on the electronic properties of our QDs and bulk GaAs is described in the main text and also in Supplementary Note 8-12.

**Supplementary Note 4: Additional photoluminescence data on macroscopic membranes**

To confirm the reliability and reproducibility of the results shown in the manuscript (Fig. 3), measurements on another device with a large membrane were performed. The data collected on a randomly chosen QD located in the area of the membrane suspended above the actuator gap are shown in Supplementary Figure 4 Supplementary Figure 4(a) shows the color-coded PL spectra of the selected QD as a function of linear-polarization direction with no applied voltage to the actuator ($F_{\mathrm{p}}$ = 0 kV/cm). In spite of the fact that no stress is intentionally applied to the membrane, we see slightly polarized emission from the neutral exciton (X) and multiexcitonic lines (MX) and a substantially larger X fine-structure-splitting (FSS) compared to the values obtained on as-grown samples. We attribute these two observations to residual (anisotropic, see below) stress arising from the device processing and from the different thermal expansion coefficients of the materials present in the device (PMN-PT, semiconductor, SU8 etc.), as mentioned in the main text. Under strong tension, see Supplementary Figure 4(b), all lines are nearly 100% polarized, similar to the results shown in Fig. 3(c) of the main text. In Supplementary Figure 4(c), we plot the degree of linear polarization $P$ of X and MX as a function of $F_{\mathrm{p}}$ (tensile stress). For the MX lines we integrated the intensity of all lines. It is obvious that $P$ increases with the increasing tensile stress, which is consistent with the experimental and theoretical analysis in the main text.

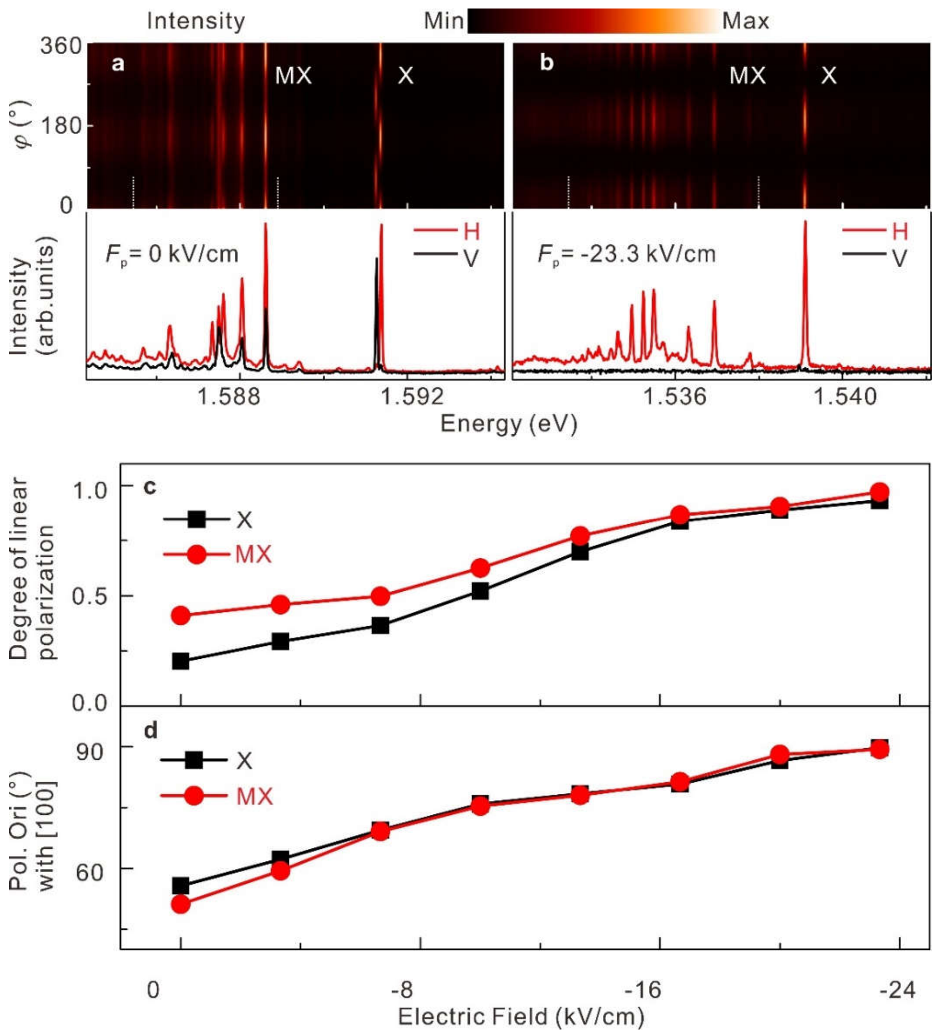


**Supplementary Figure 4**. (a-b) Color coded linear-polarization-resolved PL spectra of a GaAs QD at 0 applied field across the actuator and under high tensile stress. X and MX stand for neutral exciton and multiexcitonic lines. The slight polarization anisotropy observed in (a) is attributed to process- and cooling- induced stress. (c) Evolution of polarization degree with increasing tensile stress (magnitude of electric field $F_p$). (d) Evolution of the polarization orientation of X and MX for increasing $|F_p|$. While the initial polarization is randomly oriented, light becomes fully polarized parallel to the $y$ direction (perpendicularly to the pulling direction) at large stress.

The presence anisotropic prestress combined with random fluctuations in the confinement potential defined by the QD lead to emission which is partially linearly polarized along a random direction. In addition, the transition dipoles for the X emission have random orientation

even in absence of prestress[2,4]. In Supplementary Figure 4(d) we plot the polarization direction $\varphi^*$ along which the emission intensity is maximal as a function of the electric field across the actuator fingers (which we expect to be approximately proportional to the applied uniaxial stress). Independent on the initial polarization direction and the initial orientation of the X-dipoles, the emission lines (X and MX) become fully polarized perpendicular to the pulling direction defined by the actuator at large uniaxial stress, which is consistent with the theoretical prediction in main text.

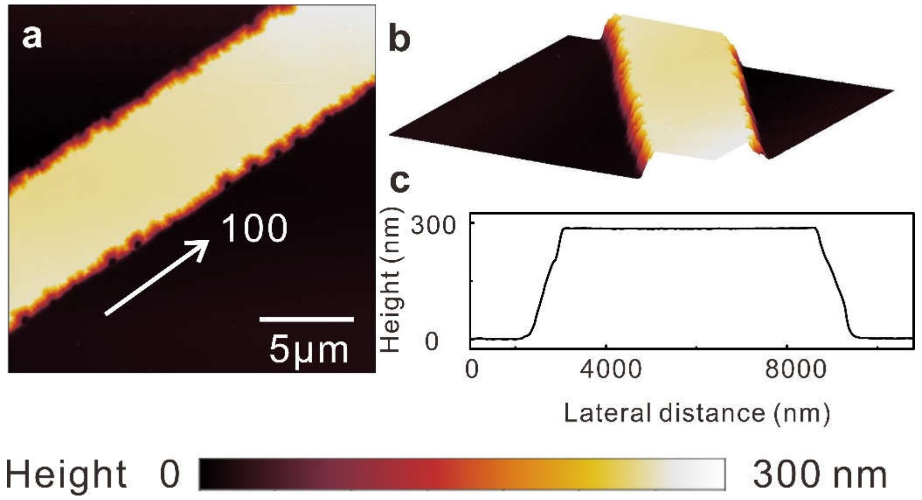


**Supplementary Figure 5**. (a) AFM image of stripe-like membrane after $H_2SO_4$:$H_2O_2$:$H_2O$ etching (b) 3D view of the AFM image of etched membrane. (c) Line profile of etched membrane.

**Supplementary Note 5: AFM topographic image of stripe membranes**

The etching solution used to produce mesa structures on the semiconductor sample consists of $H_2SO_4$:$H_2O_2$:$H_2O$ (1:8:200 in volume ratios). Etching was performed at room temperature (RT). This etching solution is widely used for GaAs[9] and is slightly anisotropic. During etching, the photoresist is undercut, leading to tilted side-walls, as illustrated by the AFM image in Supplementary Figure 5(a) and (b) and corresponding line scan in Supplementary Figure 5(c). The membrane is bonded upside-down on the actuator, so that the tilted edges act as rough reflectors, which allow us to collect $z$-polarized light from the sample top. Because of the poorly defined geometry and roughness, a quantitative evaluation of the relative intensity of $y$- and $z$-polarized components is not possible using this simple strategy.

However we expect $z$-polarized light to be "converted" in $y$-polarized light when detected from the top of the stripe, consistent with the results shown in Supplementary Figure 6 and Fig. 4 of the main text.

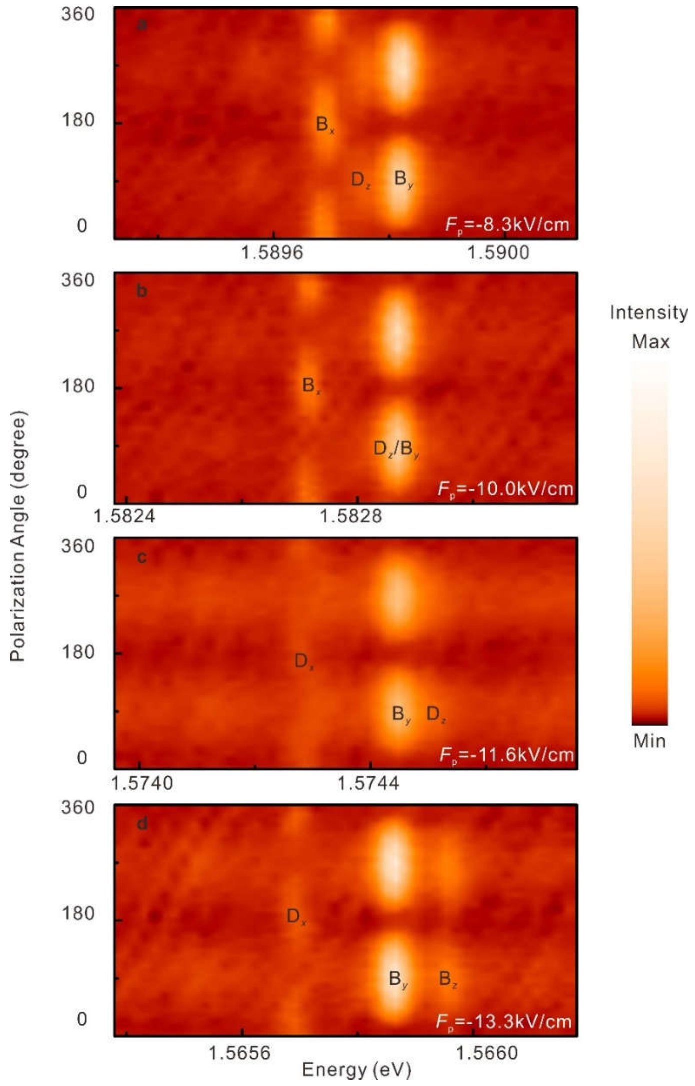


**Supplementary Figure 6**. Color-coded polarization-resolved PL signal of a neutral exciton emission in a GaAs QD for different values of the electric field applied to the actuator. Tensile stress increases from top to bottom. An angle of 0° corresponds to polarization along the pulling direction $x$. $D_z$ stands for one of the initially dark states, while $D_x$ is the dark exciton state under tensile stress, $B_y$ and $B_z$ are the new two bright exciton states under tensile stress. The chosen color-scale enhances also weak features.

**Supplementary Note 6: Linear-polarization-resolved PL spectra of neutral excitons confined in QDs embedded in stripe membranes**

Supplementary Figure 6 shows a set of polarization-resolved PL spectra of a neutral exciton X confined in a GaAs QD (QD1) under different tensile stresses [the same dot shown in Figure 2(c) of the main text]. These spectra provide a clear picture of the evolution of the X fine structure during the transition from a $HH_z$ to a $HH_x$ hole ground state (HGS). With increasing uniaxial stress, the initially dark exciton $D_z$ becomes bright [Supplementary Figure 6(a)] and gradually moves from the low energy side of the bright exciton $B_y$ to its high energy side, and finally becomes a bright exciton $B_z$ [Figure S6(d)]. The *z*-polarized component ($D_z$ or $B_z$) is reflected by the sidewalls of stripe membrane and appears to have polarization approximately parallel to the *y* direction, as expected. The initially bright exciton $B_x$ moves to the low side energy side of $B_y$ with increasing stress. Moreover, the intensity of $B_x$ drops monotonically with increasing stress (for this reason we refer to it as $D_x$ for large tensile stress). Data shown in Supplementary Figure6 and additional collected for different values of the field $F_p$ applied to the actuators were averaged to obtain a single spectrum for each value of $F_p$. To highlight the evolution of the fine structure, such spectra were shifted along the energy axis using the $B_y$ as a reference. The result is shown in Supplementary Figure 7(a), (b). The same procedure was used for another QD (QD2) [Fig. 4(b-d) of the main text], which shows fully consistent behavior. Similar data for still another QD (QD3) are shown in Supplementary Figure 7(c), (d).

Overall, we see very similar behavior for all QDs. In addition, the high energy component $B_z$ is consistently observed only in QDs contained in narrow stripes and not in large membranes. It should be noted that - especially for QD3 - a "full darkening" of the $D_x$ line is not achieved within the applied range of $F_p$ values. We qualitatively attribute this observation to the presence of prestress with main axes away from the [100] and [010] directions, consistent with the large FSS observed at low $F_p$.

Lastly, the polarization orientation of the neutral exciton in QDs embedded in stripe membranes with increasing magnitude of electric field (uniaxial stress) is plotted in Supplementary Figure 7(e). Consistent results are obtained with QDs embedded in macroscopic

membranes: the polarization orientations show initially (no intentional uniaxial stress applied) quite random direction, and become perpendicular to the pulling direction ([100] direction) with increasing uniaxial stress. All these results provide compelling evidence that uniaxial stress can be used to deterministically set the orientation of the transition dipoles in a QD, consistent with the theory.

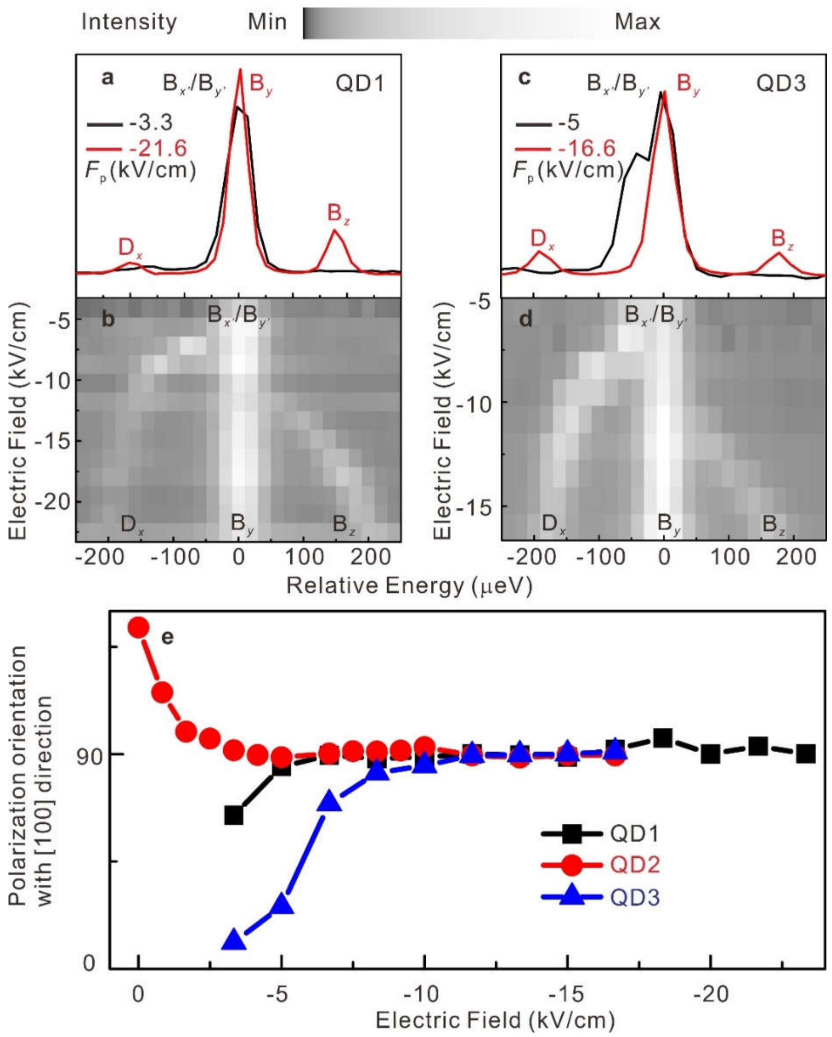


**Supplementary Figure 7**. Evolution of fine structure of the neutral exciton confined in different QDs for increasing magnitude of electric field applied (uniaxial stress). (a), (b) for QD1, (c), (d) for QD3. Similar plots for QD2 are shown in the main text. (e) Evolution of the neutral exciton polarization orientation for different QDs for varying magnitude of electric field applied to the actuator (uniaxial stress).

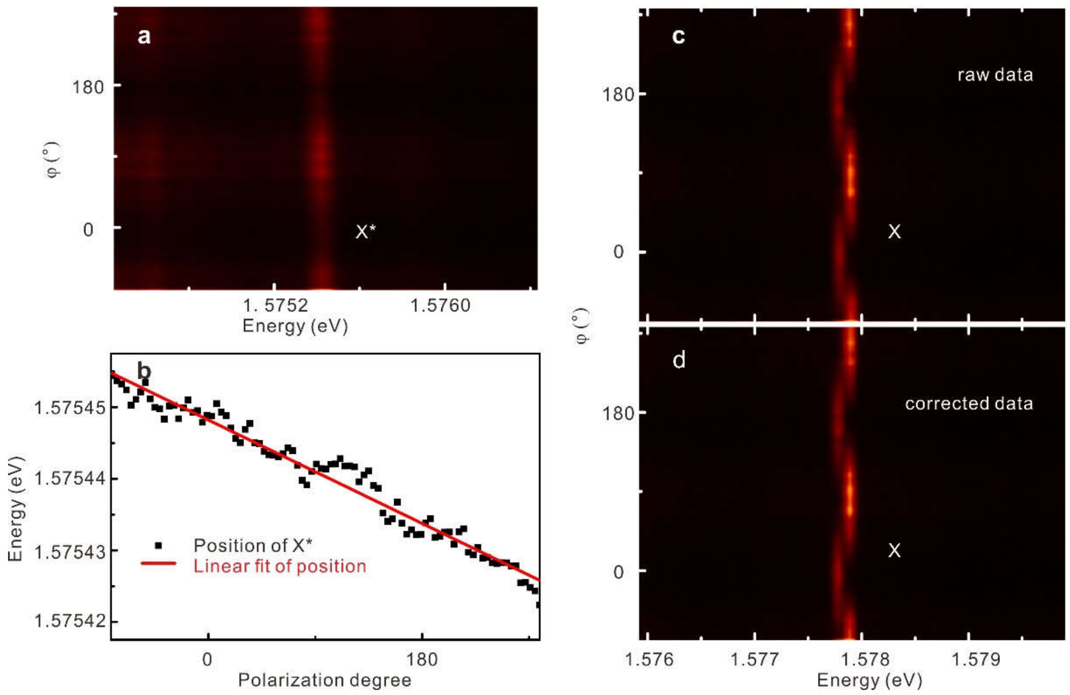


**Supplementary Figure 8**. Illustration of the procedure used to compensate piezo-creep after data acquisition. (a) Typical polarization-resolved PL spectra of a trion (marked as X*) emission. (b) Energy position of the trion emission as a function of polarization angle [obtained from Gaussian fitting of the spectra shown in (a)] and its linear fit. (c), (d) the raw and corrected polarization resolved PL spectra of the neutral exciton (marked as X) emission of the same dot.

## Supplementary Note 7: Data correction for piezo creep-compensation

Piezo-creep is a quite common phenomenon in ferroelectric materials and typically results in a logarithmically-varying strain at a fixed applied electric field[12]. As mentioned in the main text piezo-creep was observed during the measurements, especially at large $F_p$. This problem, which is not significant for small fields and can be fully compensated by an active feedback,[10,11] was addressed here by combining waiting times of about 10-20 min after setting a new piezo-voltage value for new polarization-resolved measurements and data correction after acquisition. To illustrate the procedure, we take the QD shown in Fig. 3 in the main text with applied electric field of -10 kV/cm as an example. Generally the PL signal of the trion is chosen as a reference due to its rather high intensity, narrow full-width-at-half-maximum (FWHM), and absence of fine-structure, as shown in Supplementary Figure 8(a). Then, through Gaussian curve fit of the

trion emission line, the peak position for each polarization angle is plotted, see Supplementary Figure 8(b). For sufficiently long waiting times and relatively short acquisition time for the PL data, the creep-induced shift is approximately linear. A slope of the linear fit of the peak position vs polarization angle is used to slightly shift the polarization-resolved PL spectra. Supplementary Figure 8(c), (d) show the raw and corrected polarization resolved PL spectra of neutral exciton emission, respectively.

**Supplementay Note 8: Definition of quantization axis based on the atomistic empirical-pseudopotential-method (EPM) and the configuration-interaction (CI)**

The HGS confined in the QD was calculated with the EPM+CI methods and projected to the eigenstates of the angular-momentum-projection operator $J_{\mathbf{n}}=\mathbf{J}\cdot\mathbf{n}$, with $\mathbf{n}=(\cos\vartheta, 0, \sin\vartheta)$ in the *x-z* plane for increasing tensile stress. (The eigenstates can be obtained from those of the $J_z$ operator usind the transformations provided in Supplementary Note 9). The result for the projection $|\langle HGS|HH_{\boldsymbol{n}}\rangle|^2$ is graphically shown in Supplementary Figure 9. Large values of the projection – let's say above 0.9 (a typical value of $|\langle HGS|HH_{\boldsymbol{z}}\rangle|^2$ for conventional Stranski-Krastanow QDs) can be interpreted as the indication that it is meaningful to define a quantization axis along the direction specified by **n**. Low values indicate instead mixed HH-LH states, for which the total angular momentum is not well defined. The plot clearly shows that a z quantization axis is appropriate for our as-grown QDs, while the quantization axis is oriented along the x direction for large values of strain). The data shown in red in Fig. 1**b** and 1**c** of the main text correspond to vertical scans for $\vartheta$=0 (*z*-axis) and $\vartheta$=π/2 (*x*-axis), respectively.

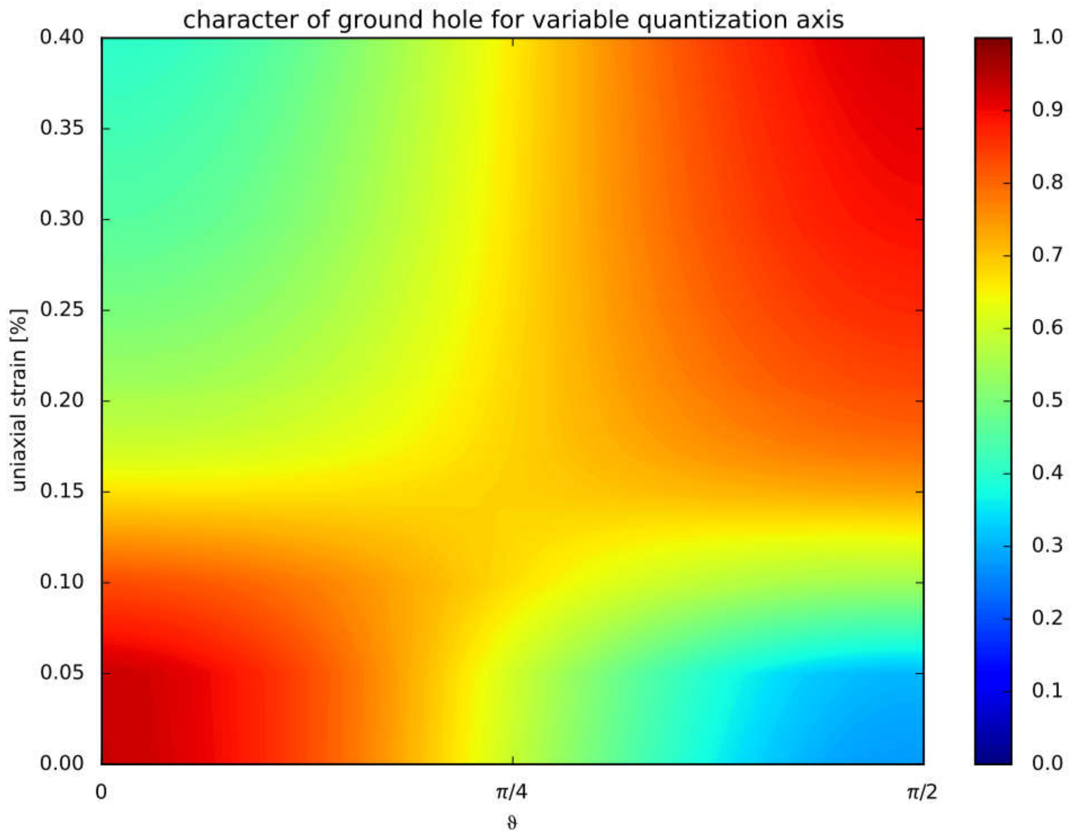


**Supplementary Figure 9.** Value of the projection of the HGS on the $HH_n$ state for different values of strain. Large values of the projection indicate that it is meaningful to define a quantization axis along the direction specified by the unit vector **n**=(cos$\vartheta$, 0, sin$\vartheta$) in the *x*-*z* plane ($\vartheta$=0 corresponds to the *z*-axis while $\vartheta$=π/2 corresponds to the *x* axis).

## Supplementary Note 9: k.p model: bulk limit

To illustrate the effect of uniaxial stress on the quantization axis of our QDs we have used **k.p** theory, which is well suited to describe the band structure of semiconductors close to a specific point of the Brillouin zone (here the zone center, i.e. the Γ point).

Since the CB is energetically well separated from the VBs, we have neglected coupling and treated separately the CB and the VBs. The dispersion of the 6 VBs and the effect of strain are described by the Luttinger-Kohn and Pikus-Bir Hamiltonians. For the periodic part of the Bloch wave functions $|u_i\rangle, i = 0 \dots 8$ for electrons in the CB and holes in the HH, LH, SO bands at the Γ point we use the convention of Ref.[18]:

$|u_{CB}^{1}\rangle = iS \uparrow;$ $|u_{CB}^{2}\rangle = iS \downarrow$

$|u_{HH}^{1}\rangle = -\frac{1}{\sqrt{2}}(X + iY) \uparrow$ ;

$|u_{LH}^{1}\rangle = -\frac{1}{\sqrt{6}}[(X + iY) \downarrow -2Z \uparrow];$ $|u_{LH}^{2}\rangle = \frac{1}{\sqrt{6}}[(X - iY) \uparrow +2Z \downarrow]$ (4)

$|u_{HH}^{2}\rangle = \frac{1}{\sqrt{2}}(X - iY) \downarrow$

$|u_{SO}^{1}\rangle = \frac{1}{\sqrt{3}}[(X + iY) \downarrow +Z \uparrow];$ $|u_{SO}^{2}\rangle = \frac{1}{\sqrt{3}}[(X - iY) \uparrow -Z \downarrow]$

Here *X, Y, Z* have the same angular dependence as $(\cos\varphi\ \sin\theta, \sin\varphi\ \sin\theta, \cos\theta)$ and can be expressed as proper linear combination of the spherical harmonics with angular momentum quantum number *l*=1 (see Ref. [18]) and S is spherically symmetric (*l*=0). The vertical arrows stand with spin up and spin down states with respect to the z quantization axis. In the main text we have called the *X, Y, Z* states as $|X_i\rangle$. By using this basis, the Hamiltonian reads:

$$\mathrm{H} = -\begin{pmatrix} -C & 0 & 0 & 0 & 0 & 0 & 0 & 0 \\ 0 & -C & 0 & 0 & 0 & 0 & 0 & 0 \\ 0 & 0 & P+Q & -S & R & 0 & -\frac{S}{\sqrt{2}} & \sqrt{2}R \\ 0 & 0 & -S^{+} & P-Q & 0 & R & -\sqrt{2}Q & \sqrt{\frac{3}{2}}S \\ 0 & 0 & R^{+} & 0 & P-Q & S & \sqrt{\frac{3}{2}}S^{+} & \sqrt{2}Q \\ 0 & 0 & 0 & R^{+} & S^{+} & P+Q & -\sqrt{2}R^{+} & -\frac{S^{+}}{\sqrt{2}} \\ 0 & 0 & -\frac{S^{+}}{\sqrt{2}} & -\sqrt{2}Q^{+} & \sqrt{\frac{3}{2}}S & -\sqrt{2}R & P+\Delta & 0 \\ 0 & 0 & \sqrt{2}R^{+} & \sqrt{\frac{3}{2}}S^{+} & \sqrt{2}Q^{+} & -\frac{S}{\sqrt{2}} & 0 & P+\Delta \end{pmatrix} \quad (5)$$

with:

$$C = E_v + E_g + C_k + C_\varepsilon, \quad C_k = \frac{\hbar^2 k^2}{2m^*}, \quad C_\varepsilon = a_c(\varepsilon_{xx} + \varepsilon_{yy} + \varepsilon_{zz})$$

$$P = E_v + P_k + P_\varepsilon, \quad P_k = \frac{\hbar^2 k^2}{2m}\gamma_1, \quad P_\varepsilon = -a_v(\varepsilon_{xx} + \varepsilon_{yy} + \varepsilon_{zz})$$

$$Q = Q_k + Q_\varepsilon, \quad Q_k = \frac{\hbar^2}{2m}\gamma_2(k^2 - 3k_z^2), \quad Q_\varepsilon = -\frac{b}{2}(\varepsilon_{xx} + \varepsilon_{yy} - 2\varepsilon_{zz})$$

$$R = R_k + R_\varepsilon, \quad R_k = \frac{\hbar^2}{2m}(\gamma_2(k_x^2 - k_y^2) + 2i\sqrt{3}\gamma_3 k_y k_x), \quad R_\varepsilon = \frac{\sqrt{3}b}{2}(\varepsilon_{xx} - \varepsilon_{yy}) - id\varepsilon_{zy}$$

$$S = S_k + S_\varepsilon, \quad S_k = \frac{\hbar^2}{2m}\gamma_3\sqrt{3}(k_x k_z - ik_y k_z), \quad S_\varepsilon = -d(\varepsilon_{xz} - i\varepsilon_{yz})$$

(6)

*x,y,* and *z* correspond to the [100], [010] and [001] crystal directions, respectively; $E_g$ is the

energy bandgap and $E_v$ the energy of the HH and LH valence bands at the Γ point in absence of strain; the parameters γ are the Luttinger parameters, $m^*$ is the electron effective mass and $a_c$, $a_v$, $b$, $d$ are the deformation potentials. The values of these parameters were taken from Ref. [19].

By diagonalizing the Hamiltonian for a fixed value of **k** we obtain 8 eigenvalues $\lambda_i$ (each at least twice degenerate) and the corresponding eigenvectors $|u_v^i\rangle$ for the valence bands (in general a mixture of HH, SO and LH with different spins) and $|u_c^j\rangle$ for the two conduction bands. In general, the eigenstates corresponding to the VBs can be written as:

$$|u_v^i\rangle = \sum_{m=0}^{8} a_m^i \;|u_m\rangle \quad (7)$$

Note that in the model used above the coefficients corresponding to the CB are zero. Since the basis states are linear combinations of the *X, Y, Z* states with spin-up and spin-down, the eigenstates of the Hamiltonian can also be expressed as a linear combination of such states, as stated in the main text.

The model can be used to calculate the band-structure in proximity of **k**=**0** as illustrated in Figs. 1(b-c) of the main text. As an additional remark we would like to add that the nature ("heavy" or "light") of the highest energy VB can be qualitatively understood in a tight-binding picture. A $HH_x$ state has only contributions from atomic orbitals lying in the *y-z* plane and no contribution from $p_x$ orbitals (similar to a $HH_z$ state having contributions from atomic orbitals in the *x-y* plane and no contribution from $p_z$ orbitals, see form of $|u_{HH}^{1,2}\rangle$ above). Upon tension along the *x* direction the overlap between the $p_x$-like atomic orbitals decreases along the "stretched" direction, weakening the bonds involved. In contrast, the overlap between the $p_y$ and $p_z$-like atomic orbitals increases (to a lesser extent than the reduced overlap because the relative displacement along *x* (strain component $\varepsilon_{xx}$) is much larger than $\varepsilon_{yy}$). A decreased/increased overlap leads to a drop/increase of the energy associated with the bond (due to Heisenberg's principle). Following this argument it is clear that the energy of the $HH_x$ state increases under tension, while that of the $LH_x$ state drops with respect to the unstrained situation. This simple picture allows us also to understand the origin of the anisotropic effective mass of the two bands. The $HH_x$ state has no $p_x$ orbitals, making the motion of electron motion along the *x* direction "difficult". The effective mass of such electrons is thus "large". In the perpendicular direction motion is instead "easy" because of the presence of $p_y$ and $p_z$ orbitals

with improved overlap. For the LH$_x$ state we have instead "easy" motion along $x$ (although with an increased effective mass compared to the unstrained case) and "difficult" in the perpendicular direction (because the LH$_x$ state consists mostly of p$_x$ orbitals). Similar arguments can be applied for the compressive case. It should be noted that the topmost band (LH$_x$) is characterized by an extremely light effective mass in this case, since compression along $x$ produces a pronounced improvement of the overlap between p$_x$ atomic orbitals).

We now provide an additional argument to illustrate that the quantization axis can be rotated by strain. For sufficiently large strain, we can neglect the effects produced by confinement. In addition, because of the relatively large value of the spin-orbit coupling-constant $\Delta$ we can limit our attention to the topmost 4 bands, which are described by the following 4×4 block of the Hamiltonian in Eq. 1:

$$H_{4\times4} = -\begin{pmatrix} P+Q & -S & R & 0 \\ -S^{+} & P-Q & 0 & R \\ R^{+} & 0 & P-Q & S \\ 0 & R^{+} & S^{+} & P+Q \end{pmatrix} \tag{8}$$

For compressive biaxial stress in the $x$-$y$ plane (which we have mentioned in the main text), the non-zero components of the strain tensor are $\varepsilon_{xx} = \varepsilon_{yy} < 0$ and $\varepsilon_{zz} = -\frac{2C_{12}}{C_{11}}\varepsilon_{xx} \sim -0.9\varepsilon_{xx} > 0$.

With the same basis used for the Hamiltonian, the operator for the $z$ component of the total angular moment of an electron in the HH or LH bands is given by:

$$\hat{J}_z = \hbar \begin{pmatrix} \frac{3}{2} & 0 & 0 & 0 \\ 0 & \frac{1}{2} & 0 & 0 \\ 0 & 0 & -\frac{1}{2} & 0 \\ 0 & 0 & 0 & -\frac{3}{2} \end{pmatrix} \tag{9}$$

It is easy to see that this operator commutes with the Hamiltonian at the $\Gamma$ point, since both $S$ and $R$ are zero in the case of $\varepsilon_{xx} = \varepsilon_{yy}$. This means that it is possible to choose the eigenstates of $H_{4\times4}$ and $\hat{J}_z$ simultaneously. In other words, the eigenstates have well defined $J_z$.

In the case of uniaxial stress along $x$ we have $\varepsilon_{zz} = \varepsilon_{yy}$ and $\varepsilon_{xx} = -\frac{C_{11}+C_{12}}{C_{12}}\varepsilon_{zz} \sim -3.2\varepsilon_{zz}$. We see from this expression, that uniaxial stress produces a strain configuration, which

is much more anisotropic than biaxial stress, as mentioned in the main text. Now $S$ is still 0 (because there is no shear strain), but $R$ does not vanish. Since the operator for the component $x$ of the total angular moment is given by:

$$\hat{J}_x = \hbar \begin{pmatrix} 0 & \frac{\sqrt{3}}{2} & 0 & 0 \\ \frac{\sqrt{3}}{2} & 0 & 1 & 0 \\ 0 & 1 & 0 & \frac{\sqrt{3}}{2} \\ 0 & 0 & \frac{\sqrt{3}}{2} & 0 \end{pmatrix} \tag{10}$$

it is easy to see that now $[\hat{J}_x,\ H_{4\times4}]=0$, i.e. that the quantization axis is parallel to the $x$ axis.

**Supplementary Note 10. Inclusion of vertical confinement and calculation of mixing**

Because of the flat morphology of the studied QDs, the main effect of confinement stems from the vertical ($z$) direction. We included the effect of vertical confinement in our **k·p** model following two approaches. The simplest is to emulate the effect of confinement by adding a fixed biaxial compression, as described in the text. After diagonalization of the Hamiltonian, the eigenstates are projected either to the (HH, LH, SO)$_z$ states or to the (HH, LH, SO)$_x$ states to obtain the degree of mixing for the $z$ or the $x$ quantization axis (shown in Fig. 1(e,f)). The (HH, LH, SO)$_x$ states are obtained from the conventional (HH, LH, SO)$_z$ states using the transformations provided in Ref. [18]:

$$\begin{pmatrix} \uparrow' \\ \downarrow' \end{pmatrix} = \begin{pmatrix} e^{-i\frac{\varphi}{2}}\cos\frac{\vartheta}{2} & e^{i\frac{\varphi}{2}}\sin\frac{\vartheta}{2} \\ -e^{-i\frac{\varphi}{2}}\sin\frac{\vartheta}{2} & e^{i\frac{\varphi}{2}}\cos\frac{\vartheta}{2} \end{pmatrix} \begin{pmatrix} \uparrow \\ \downarrow \end{pmatrix} \tag{11}$$

$$\begin{pmatrix} X' \\ Y' \\ Z' \end{pmatrix} = \begin{pmatrix} \cos\vartheta\cos\varphi & \cos\vartheta\sin\varphi & -\sin\vartheta \\ -\sin\varphi & \cos\varphi & 0 \\ \sin\vartheta\cos\varphi & \sin\vartheta\sin\varphi & \cos\vartheta \end{pmatrix} \begin{pmatrix} X \\ Y \\ Z \end{pmatrix} \tag{12}$$

with $\vartheta = 90°, \varphi = 0°$ for the $x$-axis.

For Fig. 1(d-f) we have used a biaxial compression of -120 MPa to obtain results close to those obtained by the EPM method. The Bloch wavefunctions shown in Fig. 1 were obtained by replacing the states *X, Y,* and *Z* with their representation in polar coordinates. The evolution of the Bloch wavefunction of the topmost VB states in prestressed bulk GaAs under uniaxial stress

is shown in the Supplementary Movie. This was obtained by assuming a fixed biaxial stress $\sigma_{xx} = \sigma_{yy} = -120$ MPa superimposed to a variable uniaxial stress $\sigma_{xx}$ from 0 to 320 MPa.

A more correct approach to include the vertical confinement is to consider a GaAs quantum well (QW) with thickness $h_{QW}$ embedded in $Al_{0.4}Ga_{0.6}As$ barriers. The confined states can be calculated by combining $\mathbf{k}\cdot\mathbf{p}$ theory with the effective mass equation. In this approach, $k_z$ in Eq. 1 is replaced by the operator $-i\frac{\partial}{\partial z}$ and the spatial dependence of the VB-edge energy $E_V(z)$ acts as a potential. For the VB profile we have used simple linear interpolations (Vegard's law) of the values of $E_{v,av}$ for GaAs and AlAs provided in Table C.2 of Ref.[18] while all other parameters are obtained using the Vegard's law on parameters provided in Ref.[19]. The uniaxial stress applied to the structure is varied continuously and the corresponding strain values are assumed to be constant throughout the heterostructures (we used the stiffness constants of GaAs also for the barriers). We solved the QW problem by the finite difference method. The $z$ direction is discretized in N steps and each of the elements appearing in Eq. 1 is replaced by a N×N block. To guarantee that the resulting 6N×6N matrix describing the VBs is Hermitian we used the approximations provided by Ref.[20] for the derivatives (Note that in the second term of Eq. 35 one of the $A(z_{i-1})$ should be replaced by $A(z_{i+1})$). Diagonalization is performed with the LA_EIGENVEC routine (from LAPACK) implemented in IDL6.4.

Supplementary Figure 9 shows the result for the mixing evaluated along the $z$ axis and $x$ axis for $h_{QW}$=4 and 12 nm. (Our QDs have a height between 7 and 9 nm). It is evident that the trends closely resemble those obtained with the simplified "bulk" model with a fixed prestress and the more sophisticated 3D EPM model (see Fig. 1(b-c) of main text). We note that for a QW we have a pure $HH_z$ state in absence of strain. In a lens-shaped QD this is not the case because the lateral confinement and other effects induces some LH-HH mixing, as previously calculated for the dots considered here in Refs.[17,21]. In addition we see that the transition from a $z$- to an $x$-oriented quantization axis becomes smoother when the quantum well thickness decreases. In particular we see that for a thin QW substantial mixing persists under large uniaxial tension. This means that tall QDs are required to achieve full rotation of the axis and reduce residual mixing. In addition, compressive stress present in commonly studied SK dots would further hinder the rotation of the quantization axis.

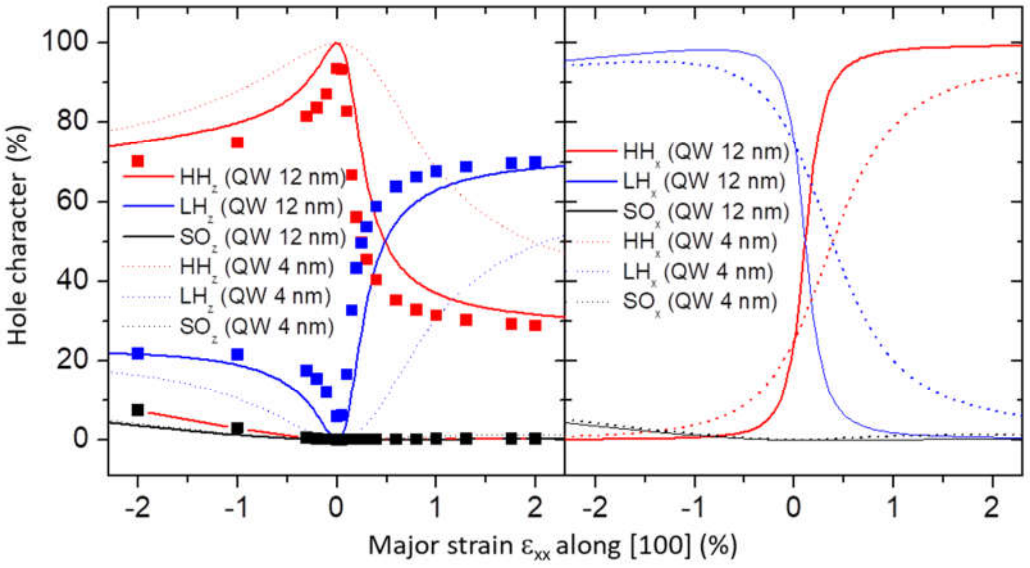


**Supplementary Figure 9.** Calculation of mixing for GaAs/AlGaAs QWs of different thickness. The rotation of the quantization axis is facilitated by a larger QW thickness. This means that tall GaAs QDs are preferable. The results of the EPM calculations shown in Fig. 1 of the main text are reported as symbols.

**Supplementary Note 11. Emission energy shift under uniaxial stress along [100]**

Since we have not determined the strain induced by our actuators experimentally, we can estimate the strain by comparing the observed energy shift to that expected from calculations. Supplementary Figure 10 shows the calculated emission energy of an exciton in a GaAs QD computed by the EPM+CI methods and a simple **k·p** calculation for QWs with thickness ranging from 4 to 12 nm (in this case no excitonic effects were included). While we have linear shifts for sufficiently large tensile strains, the behavior is strongly non-linear at small strains and in the compressive regime. This effect, which is seen both in the EPM and **k·p** calculations, stems from an anticrossing of the two uppermost VBs, as illustrated in Supplementary Fig. 11, where we have used again the bulk Hamiltonian of Eq. 1. To emulate the behaviour of a QW, we have shifted the CB terms by 52.8 meV (calculated confinement energy for electrons in a 8 nm QW), the diagonal HH terms ($P$+$Q$) by 9.1 meV and added a fixed energy shift of 10 meV to the diagonal terms corresponding to LH.

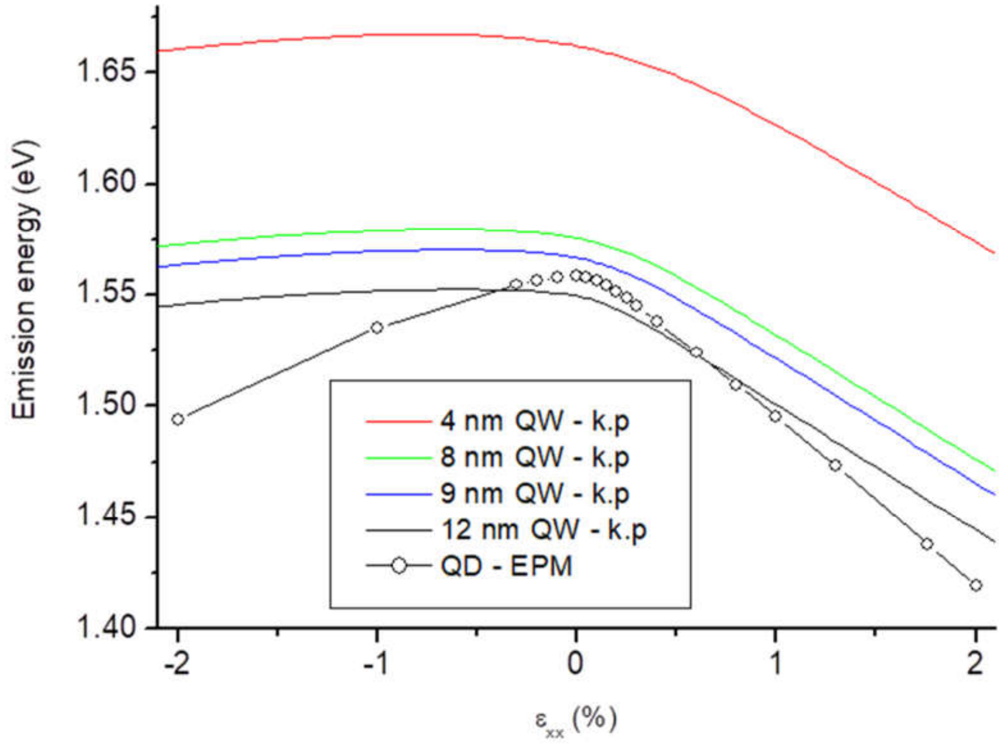


**Supplementary Figure 10.** Calculated emission energy of a GaAs QD via the EPM and CI methods and comparison with the emission energy of QWs with different thickness calculated with the **k·p** method.

The EPM and **k·p** calculations follow qualitatively the same trends. However the EPM results shows enhanced slopes both under tension and compression. Although the reason is not yet clear, we can estimate that an energy shift of 100 meV (see Fig. 2c of the main text) corresponds to a major strain of about 1.5-1.7%.

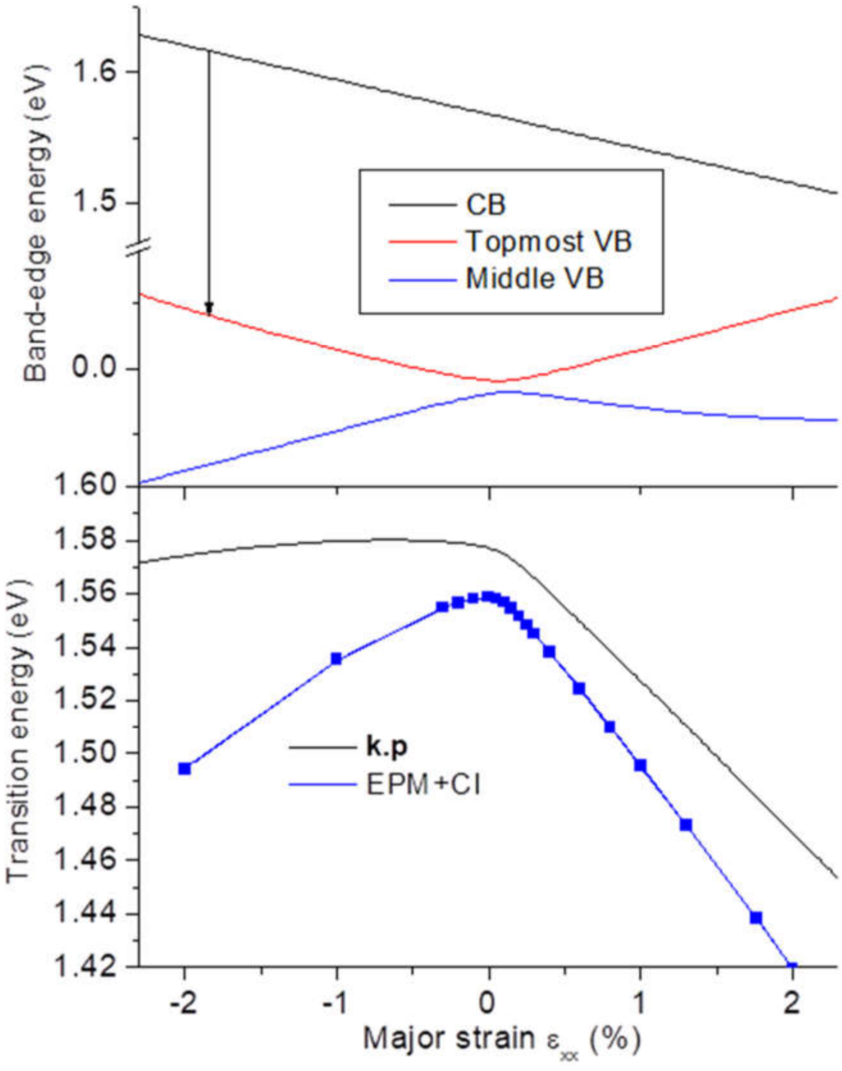


**Supplementary Figure 11.** Illustration of the origin of the non-linear energy shift of the QD emission.

**Supplementary Note 12. Evolution of selection rules under uniaxial stress**

The change of quantization axis upon uniaxial stress changes substantially the selection rules for dipole-allowed transitions. Both theoretically and experimentally we have seen that initially bright transitions turn dark and initially dark transitions turn bright.

By using the experimentally measured radiative lifetime $\tau$ of bright excitons in unstrained QDs (with $HH_z$ ground state)[22] we can estimate the transition rates $\tau^{-1}$ for transitions with polarization along the axes of the cubic cell from both the EPM and **k·p** calculations as shown in Fig. 5**a** of the main text. For the sake of completeness we show in Supplementary Figure 12 the calculated transition strengths for a QW. At 0 strain the HGS has dominant $HH_z$ character

according to the 3D EPM calculations (or pure $HH_z$ character according to the 1D calculations for a 12-nm thick QW). Under tensile strain we have the transition to a $HH_x$ HGS, which does not couple to *x*-polarized light. The transition rate for the *x*-polarized emission is slightly higher than for the initially unstrained QD, while the *z*-polarized transition is slightly weaker. In other words the overall oscillator strength, which is initially equally shared between the in-plane transition-dipoles is redistributed in a slightly asymmetric fashion under uniaxial tension. A direct comparison with the experiment would require time-resolved measurements of temporal decay.

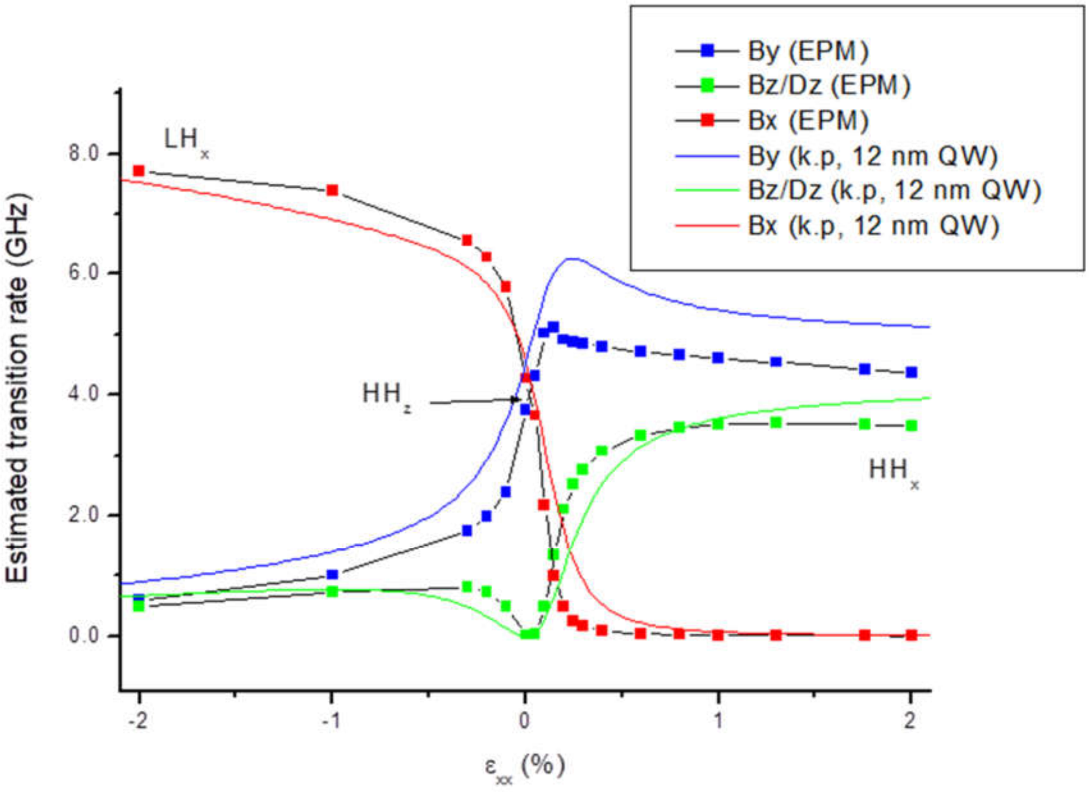


**Supplementary Figure 12.** Estimated transition rates for light polarized parallel to the *x, y,* and *z* direction.

Under compression (not investigated experimentally yet, but very interesting for integrated-photonics applications), almost the whole oscillator strength is transferred to the *x*-polarized emission. The effect is robust, as it is reproduced both by the 3D and 1D simulations and stems from the properties of bulk GaAs (shown in Fig. 5**a** of the main text).

**Supplementary References**


1. Stemmann, A., Heyn, C., Köppen, T., Kipp, T. & Hansen, W. Local droplet etching of nanoholes and rings on GaAs and AlGaAs surfaces. *Appl. Phys. Lett.* **93,** 12–15 (2008).

2. Huo, Y. H., Rastelli, A. & Schmidt, O. G. Ultra-small excitonic fine structure splitting in highly symmetric quantum dots on GaAs (001) substrate. *Appl. Phys. Lett.* **102,** 152105 (2013).

3. Rastelli, A. *et al.* Hierarchical self-assembly of GaAs/AlGaAs quantum dots. *Phys. Rev. Lett.* **92,** 166104–1 (2004).

4. Huo, Y. H., Křápek, V., Rastelli, A. & Schmidt, O. G. Volume dependence of excitonic fine structure splitting in geometrically similar quantum dots. *Phys. Rev. B* **90,** 041304 (2014).

5. Martín-Sánchez, J. *et al.* Reversible Control of In-Plane Elastic Stress Tensor in Nanomembranes. *Adv. Opt. Mater.* **4,** 682–687 (2016).

6. Trotta, R. *et al.* Wavelength-tunable sources of entangled photons interfaced with atomic vapours. *Nat. Commun.* **7,** 10375 (2016).

7. Cottam, R. I. & Saunders, G. a. The elastic constants of GaAs from 2 K to 320 K. *J. Phys. C Solid State Phys.* **6,** 2105–2118 (2001).

8. Herklotz, A. *et al.* Electrical characterization of PMN-28%PT(001) crystals used as thin-film substrates. *J. Appl. Phys.* **108,** (2010).

9. Tsang, W. T. & Wang, S. Profile and groove-depth control in GaAs diffraction gratings fabricated by preferential chemical etching in H2SO4-H 2O2-H2O system. *Appl. Phys. Lett.* **28,** 44–46 (1976).

10. Rastelli, A. *et al.* Controlling quantum dot emission by integration of semiconductor nanomembranes onto piezoelectric actuators. *Phys. status solidi* **249,** 687–696 (2012).

11. Trotta, R. *et al.* Nanomembrane quantum-light-emitting diodes integrated onto

piezoelectric actuators. *Adv. Mater.* **24,** 2668–2672 (2012).

12. Jung, H. & Gweon, D. G. Creep characteristics of piezoelectric actuators. *Rev. Sci. Instrum.* **71,** 1896–1900 (2000).

13. Williamson, A. J., Wang, L. W. & Zunger, A. Theoretical interpretation of the experimental electronic structure of lens-shaped self-assembled InAs/GaAs quantum dots. *Phys. Rev. B* **62,** 12963–12977 (2000).

14. Wang, L.-W. & Zunger, A. Linear combination of bulk bands method for large-scale electronic structure calculations on strained nanostructures. *Phys. Rev. B* **59,** 15806–15818 (1999).

15. Franceschetti, A., Fu, H., Wang, L. W. & Zunger, A. Many-body pseudopotential theory of excitons in InP and CdSe quantum dots. *Phys. Rev. B* **60,** 1819–1829 (1999).

16. Bester, G. Electronic excitations in nanostructures: an empirical pseudopotential based approach. *J. Phys. Condens. Matter* **21,** 023202 (2009).

17. Huo, Y. H. *et al.* A light-hole exciton in a quantum dot. *Nat. Phys.* **10,** 46–51 (2014).

18. Chuang, S. L. *Physics of photonic devices*. (John Wiley & Sons, 2009).

19. Vurgaftman, I., Meyer, J. R. & Ram-Mohan, L. R. Band parameters for III–V compound semiconductors and their alloys. *J. Appl. Phys.* **89,** 5815–5875 (2001).

20. Chuang, S. L. & Chang, C. S. A band-structure model of strained quantum-well wurtzite semiconductors. *Semicond. Sci. Technol.* **12,** 252–263 (1997).

21. Huo, Y. H., Křápek, V., Schmidt, O. G. & Rastelli, A. Spontaneous brightening of dark excitons in GaAs/AlGaAs quantum dots near a cleaved facet. *Phys. Rev. B* **95,** 165304 (2017).

22. Huber, D. *et al.* Highly indistinguishable and strongly entangled photons from symmetric GaAs quantum dots. *Nat. Commun.* **8,** 15506 (2017).
