# Supplementary material for: Uniaxial stress flips the natural quantization axis of a quantum dot for integrated quantum photonics
Source: Nat Commun. 2018 Aug 3;9:3058. doi: 10.1038/s41467-018-05499-5 (PMC6076237; doi:10.1038/s41467-018-05499-5)
Supplement: Supplementary file 3 — Description of Additional Supplementary Files [file 41467_2018_5499_MOESM3_ESM.pdf]

Description of Additional Supplementary Files

File Name: Supplementary Movie 1
Description: Evolution of the angular dependence of the Bloch wavefunction of the topmost VB states in biaxially prestressed bulk GaAs under variable uniaxial tension. The initial "donut" is characteristic of a conventional heavy-hole state and is invariant for rotations about the z-axis. Under uniaxial tension, the shape resembles a slightly squeezed "donut" with symmetry axis along the pulling direction. Further information is provided in the Supplementary Note 10.
